# P-557. Weight Change After Switching from Bictegravir/emtricitabine/tenofovir alafenamide in Persons Living with HIV: A Retrospective Cohort Study

**DOI:** 10.1093/ofid/ofae631.756

**Published:** 2025-01-29

**Authors:** Hilal Abdessamad, Margaret Kurop, Jane McKelvy, Dima Dandachi

**Affiliations:** University of Missouri, Columbia, Missouri; University of Missouri, Columbia, Missouri; University of Missouri, Columbia, Missouri; University of Missouri - Columbia, Columbia, Missouri

## Abstract

**Background:**

Antiretroviral Therapy (ART) has revolutionized the management and outcomes of persons with HIV (PWH). However, ART has undesirable effects such as weight gain, that lead to non-compliance or medication switch. The combination Bictegravir (BIC)/emtricitabine (FTC)/tenofovir alafenamide (TAF) has been associated with weight gain, though most of the data regarding this is not comprehensive.

**Figure 1:**
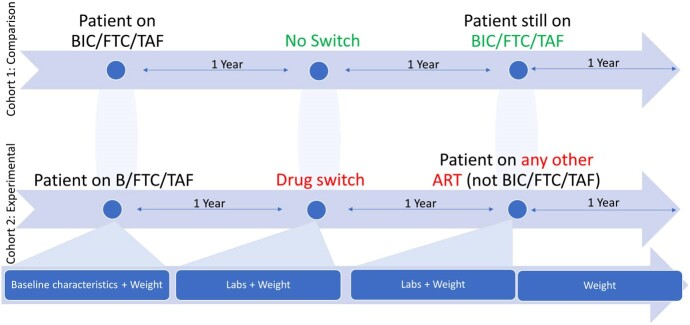
Study Flowchart

**Methods:**

A retrospective cohort study was performed on 125 patient records to assess the changes in weight, lipid panel and glycosylated hemoglobin (HBA1C) measurements in PWH who were taking BIC/FTC/TAF for at least 1 year then switched to a different combination, compared to those who remained on this regimen, over a period of 3 years (Figure 1).

Table 1

**Figure:**
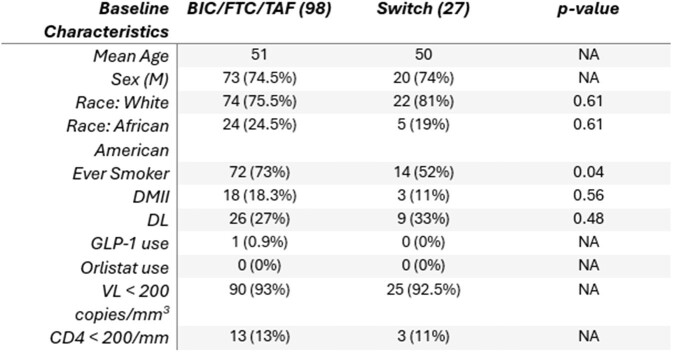

M: Male, DMII: Type 2 Diabetes Mellitus, DL: Dyslipidemia, GLP-1: Glucagon-like Peptide-1, VL: Viral Load, NA: Not Applicable

**Results:**

Out of the 125 records reviewed, 98 (78%) continued BIC/FTC/TAF for 3 years and 27 (22%) were switched to another combination after 1 year, whether cabotegravir/rilpivrine (69%), dolutegravir/lamivudine (18%) or others (13%). The 2 groups had similar baseline mean age, sex, race, metabolic profile, viral load and CD4 counts (Table 1). There was a median weight gain of 1.3 kg after 1 year of treatment with BIC/FTC/TAF. By year 2, there was a median weight loss of 0.8kg in the group who continued the same ART and 0.2 kg in the group who switched treatment (p=0.441). However, by year 3, there was a median weight gain of 0.5 kg in those who continued the same ART and 0.2 kg in those who switched to another combination; p=0.477 (Figure 2). 25/98 (25%) patients who kept same ART experienced weight loss after 2 years, in comparison to 9/26 (34%) of patients who switched treatment (p=0.497). Low-density lipoprotein and HBA1C readings had minimal mean variations between the 2 groups.

**Figure 2:**
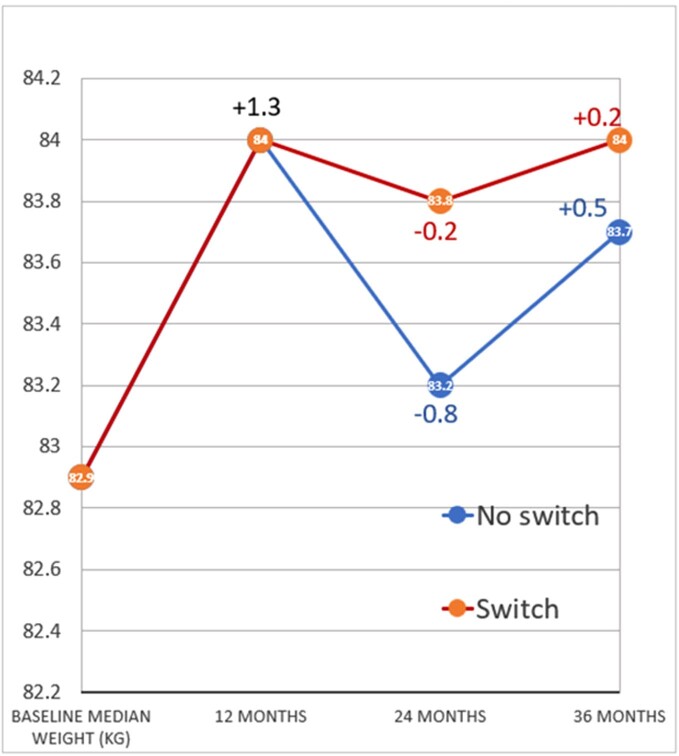
Change in median weight between the two patient groups after a 3-year follow-up.

**Conclusion:**

Our data, although of small sample size, shows that while there appears to be no significant weight change while continuing BIC/FTC/TAF as compared to switching to a different regimen. Hence, switching to treatment combinations did not result in weight loss or lower LDL and HBA1C levels. To our knowledge, this is among the few studies to provide real world data on weight change after switching from B/FTC/TAF, which highlights its significance.

**Disclosures:**

**Dima Dandachi, MD, MPH**, Gilead Sciences: Grant/Research Support|ViiV Healthcare: Grant/Research Support|ViiV Healthcare: Honoraria

